# The Role of Extracorporeal Blood Purification in the Treatment of a Patient with Lemierre's Syndrome

**DOI:** 10.1155/2022/8522398

**Published:** 2022-05-28

**Authors:** Lana Maričić, Damir Mihić, Tajana Turk, Domagoj Loinjak, Vedran Zubčić

**Affiliations:** ^1^Department of Internal Medicine, University Hospital Osijek, J.Huttlera 4, Osijek, Croatia; ^2^Faculty of Medicine Osijek, University J.J. Strossmayera Osijek, J.Huttlera 4, Osijek, Croatia; ^3^Department of Diagnostic and Interventional Radiology, University Hospital Osijek, J.Huttlera 4, Osijek, Croatia; ^4^Department of Maxillofacial and Oral Surgery, University Hospital Osijek, J.Huttlera 4, Osijek, Croatia

## Abstract

Lemierre's syndrome refers to the septic thrombophlebitis of the internal jugular vein. The condition typically begins with an oropharyngeal infection and frequently involves inflammation within the wall of the vein, infected thrombus within the lumen, surrounding soft tissue inflammation, persistent bacteremia, and septic emboli. Lemierre's syndrome is a rare disease; it occurs most commonly in otherwise healthy young adults. The most common etiologic agent is Fus*obacterium necrophorum*. We present a case of Lemierre's syndrome in a young girl and the role of extracorporeal method of blood purification with continuous venous hemodiafiltration with the use of a highly adsorptive membrane (AN69 HeprAN), thus achieving the combined elimination of cytokines and endotoxins. The use of advanced methods, along with an antibiotic and surgical treatment, will certainly help reduce mortality in this syndrome.

## 1. Introduction

Lemierre's syndrome is characterized by the thrombosis of the internal jugular vein that develops following an oropharyngeal infection. Sepsis and septic metastases frequently affect the lungs, the musculoskeletal system, and, occasionally, the liver. The most common offending micro-organism is *Fusobacterium necrophorum*. Traditional treatment modalities used are antimicrobial, anticoagulant, and surgical treatment. A beta-lactamase-resistant beta-lactam antibiotic is recommended as an empiric therapy. Antibiotics should be tailored to the culture results and susceptibility data when available. Alternative options include clindamycin or metronidazole for patients with significant clinical allergy to beta-lactams. Morbidity is significant with prolonged hospitalization in the majority of patients. The overall mortality rate is 5% [1]. In 1936, Lemierre described a syndrome that was characterized by anaerobic septicemia, internal jugular vein thrombosis, and septic emboli that are secondary to infections of the head and neck [2]. Lemierre is not the first to describe the syndrome, but because of his contribution to presentation in the literature, the syndrome was named after him. Lemierre's syndrome is basically a septic condition, and it is associated with the development of a systemic inflammatory response and with the development of multiorgan failure and poorer clinical outcomes [3]. Today, we increasingly talk about the concept of extracorporeal blood buffering as an additional method of treating sepsis, which contributes to the reduction of the hyperinflammatory response and the associated multiorgan dysfunction by eliminating fused cytokines and endotoxins [4]. We present a case of Lemierre's syndrome in a young girl and the role of extracorporeal method of blood purification with continuous venous hemodiafiltration (CVVHDF) with the use of a highly adsorptive membrane, thus achieving a combined elimination of cytokines and endotoxins. This case describes for the first time the use of the method, CVVHDF with a highly adsorptive membrane, in addition to antibiotic therapy, in order to control the inflammatory response and reduce the further development of multiorgan injury.

## 2. Case Presentation

**Figure 1:**
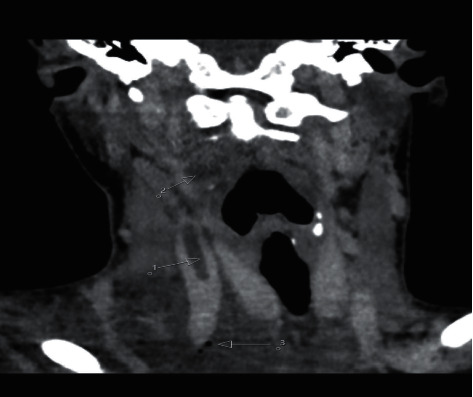
Neck computed tomography scan, coronal reconstruction, shows a thrombus in the right internal jugular vein (1), hypodense fluid collection in the ipsilateral parapharyngeal space (2), and gas inclusions in the supraclavicular region (3).

**Figure 2:**
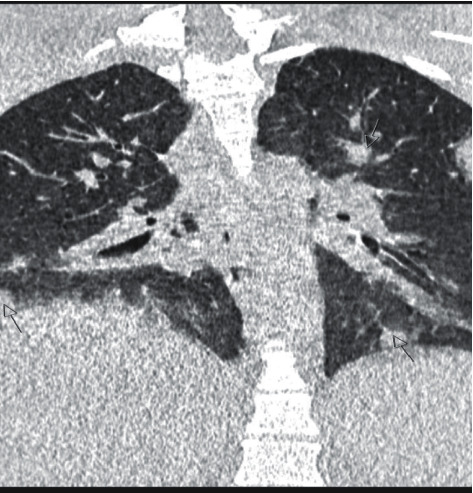
Chest computed tomography scan, coronal reconstruction, shows multiple bilateral nodular pulmonary consolidations (arrows).

A 19-year-old female without a significant past medical history was urgently admitted to hospital in a serious general condition, presenting with multiorgan failure. The infection with SARS-CoV-2 was ruled out upon admission. She was treated with phenoxymethylpenicillin on an outpatient basis for acute tonsillopharyngitis 3 weeks earlier. 2-3 days before admission, she had a fever up to 40°C, with pain in the right shoulder and right side of the chest, felt a shortness of breath on exertion, and had a dry cough. Upon admission to the hospital, she presented with fever and was hypodynamic, pale, hypotensive, and oligoanuric. The vital signs showed a low blood pressure of 80/60 mmHg, and there was a swelling of the right side of the neck and the right arm. The initial chest X-ray showed subcutaneous emphysema of the right shoulder and hemithorax, bilateral inflammatory infiltrates, and pleural effusions. Computed tomography (CT) of the neck, chest, and abdomen showed a right parapharyngeal abscess and right jugular vein thrombosis (Figure 1). Gas inclusions and fluid collection were visible in the upper mediastinum accompanied with reactive mediastinal lymphadenopathy, edematous adipose tissue, bilateral pulmonary consolidations, and hepatosplenomegaly (Figure 2). Blood tests revealed an elevated white blood cell count (24.0 × 10^9^/L), anemia (hemoglobin level: 82 g/L; hematocrit level: 0.266), and renal failure (blood urea nitrogen: 34.7 mmol/L; creatinine level: 138 *μ*mol/L). The C-reactive protein (CRP) level was also elevated at 190.2 mg/L, as well as other parameters of acute inflammation like interleukin-6 (10700.0 ng/L), ferritin (240.7 *μ*g/L), and procalcitonin (PCT) (3.83 *μ*g/L). On the same day, an emergency surgery was performed: a total exploration of the complete retropharyngeal, parapharyngeal, and retroesopharyngeal space to the depth of the aortic arch was performed. On the second day of hospitalization, due to a persistent increase in the inflammatory parameters, the CVVHDF method was performed with the adsorptive membrane (AN69 HeprAN) for 72 h. *Fusobacterium necrophorum* was isolated by a microbiological analysis of an intraoperative sample. Four days later, a surgical revision of the operative area of the neck was performed due to additional surgical debridement of the wound under general anesthesia. A follow-up blood test after the start of the CVVHDF method with the appropriate membrane showed decline in the inflammatory parameters with the CRP value of 126 mg/L, interleukin-6 of 0.12 ng/L, and PCT of 0.41 *μ*g/L. On the seventh day of hospitalization, the inflammatory parameters further declined and the follow-up CT showed regression of previously described inflammatory changes. The patient was extubated and showed a stable respiratory rate, breathing through the tracheal cannula. Since the admission of the patient, an empiric antibiotic therapy was started with intravenous meronem, with subsequent introduction of linezolid and metronidazole, as well as the anticoagulant therapy of low-molecular-weight heparin (enoxaparin). Antibiotic therapy was used for a total of up to 6 weeks. After hospital discharge, the anticoagulant therapy was continued with rivaroxaban for another 4 weeks; after a follow-up neck and chest CT confirmed complete recanalization of the right jugular vein, this was excluded from the therapy.

## 3. Discussion

A clear clinical presentation of Lemierre's syndrome after the performed diagnostic tests is in line with the previous research [5]. Our diagnosis was based on a triass of a recent pharyngeal illness, complicated by septic emboli, and the internal jugular vein thrombosis. Subsequent microbiological analysis served as the confirmation of *Fusobacterium necrophorum* infection, which represents 30% of all causes of Lemierre's syndrome. *Fusobacterium necrophorum* is an obligate anaerobic bacterium and is notoriously difficult to culture, requiring a longer incubation period than the other bacteria [6]. In the case of our patient, although treatment with antibiotic therapy was initiated immediately following the surgery, a blood purification procedure was performed on the second day, which was not stated as a common procedure in the treatment of Lemierre's syndrome. So far, a case of need for renal replacement therapy has been described in the literature [6]. In this case, in addition to established treatment methods that include surgery and antimicrobial therapy, we decided to include the extracorporeal blood purification with regard to pronounced laboratory signs of hyperinflammatory response, in order to prevent the multiorganic failure caused by systemic inflammatory response. As a method of blood purification, we used CVVHDF with the use of adsorptive membrane (AN69 HeprAN), which was primarily founded for the treatment of Gram-negative sepsis [7]. This treatment approach has already been described in other case reports of patients with Gram-negative sepsis [8]. Moreover, our use of CVVHDF with the adsorptive membrane (AN69 HeprAN) for 72 h led to the significant decrease in inflammatory factor levels. Interleukin-6 played an important role in monitoring the effect of CVVHDF, as it has been shown in our case. Because the antibiotic therapy killed the bacteria and the adsorptive membrane (AN69 HeprAN) removed the endotoxin and inflammatory mediators, this led to the reduction of the inflammation and allowed recovery from this acute illness. Although the use of this method did not shorten the length of hospitalization in this patient, but in the initial phase of treatment, the elimination of inflammatory mediators reduced the repercussions of hyperinflammatory responses. It is important to emphasize that CVVHDF with the adsorptive membrane was applied immediately at the beginning of treatment before we had a targeted antibiotic treatment.

## 4. Conclusion

Lemierre's syndrome is a rare illness in the modern era of antibiotic therapy which occurs primarily in young, otherwise healthy individuals. It is characterized by the presence of recent oropharyngeal infection, clinical or radiological evidence of venous thrombosis internal jugular vein, and the presence of anaerobic bacteremia caused primarily by *Fusobacterium necrophorum*. Prolonged antibiotic and anticoagulation therapy is the basis of the treatment. In a critical patient with a septic shock, as part of Lemierre's syndrome, it is an important adjunctive consideration to supplement the definitive source control and antimicrobial therapy. The use of extracorporeal blood purification in this case proved to be extremely important in preventing the consequences, such as multiorganic injury and faster recovery of the patient.

## References

[1] Karkos P. D., Asrani S., Karkos C. D. (2009). Lemierre’s syndrome: a systematic review. *The Laryngoscope*.

[2] Lemierre A. (1936). On certain septicaemia due to anaerobic organisms. *Lancet*.

[3] Bosmann M., Ward P. A. (2013). The inflammatory response in sepsis. *Trends in Immunology*.

[4] Monard C., Rimmelé T., Ronco C. (2019). Extracorporeal blood purification therapies for sepsis. *Blood Purification*.

[5] Johannesen K., Bodtger U. (2016). Lemierre & rsquo;s syndrome: current perspectives on diagnosis and management. *Infection and Drug Resistance*.

[6] Johannesen K., Dessau R., Heltberg O., Bodtger U. (2016). Bad news itself or just the messenger? The high mortality of Fusobacterium spp. infections is related to disseminated malignancy and other comorbidities. *European Clinical Respiratory Journal*.

[7] Nemakayala D. R., P Rai M., Kavuturu S., Rayamajhi S. (2018). Atypical presentation of lemierre’s syndrome causing septic shock and acute respiratory distress syndrome. *Case Reports in Infectious Diseases*.

[8] Schwindenhammer V., Girardot T., Chaulier K. (2019). oXiris® use in septic shock: experience of two French centres. *Blood Purification*.

